# Path-BigBird: An AI-Driven Transformer Approach to Classification of Cancer Pathology Reports

**DOI:** 10.1200/CCI.23.00148

**Published:** 2024-02-27

**Authors:** Mayanka Chandrashekar, Isaac Lyngaas, Heidi A. Hanson, Shang Gao, Xiao-Cheng Wu, John Gounley

**Affiliations:** ^1^ Advanced Computing for Health Sciences Section, Computational Sciences and Engineering Division, Oak Ridge National Laboratory, Oak Ridge, TN; ^2^ Advanced Computing for Life Science & Engineering, National Center for Computational Sciences, Oak Ridge National Laboratory, Oak Ridge, TN; ^3^ Louisiana Tumor Registry, School of Public Health, Louisiana State University Health Sciences Center, New Orleans, LA; ^4^ Department of Epidemiology, School of Public Health, Louisiana State University Health Sciences Center, New Orleans, LA

## Abstract

**PURPOSE:**

Surgical pathology reports are critical for cancer diagnosis and management. To accurately extract information about tumor characteristics from pathology reports in near real time, we explore the impact of using domain-specific transformer models that understand cancer pathology reports.

**METHODS:**

We built a pathology transformer model, Path-BigBird, by using 2.7 million pathology reports from six SEER cancer registries. We then compare different variations of Path-BigBird with two less computationally intensive methods: Hierarchical Self-Attention Network (HiSAN) classification model and an off-the-shelf clinical transformer model (Clinical BigBird). We use five pathology information extraction tasks for evaluation: site, subsite, laterality, histology, and behavior. Model performance is evaluated by using macro and micro *F*_1_ scores.

**RESULTS:**

We found that Path-BigBird and Clinical BigBird outperformed the HiSAN in all tasks. Clinical BigBird performed better on the *site* and *laterality* tasks. Versions of the Path-BigBird model performed best on the two most difficult tasks: *subsite* (micro *F*_1_ score of 72.53, macro *F*_1_ score of 35.76) and *histology* (micro *F*_1_ score of 80.96, macro *F*_1_ score of 37.94). The largest performance gains over the HiSAN model were for *histology*, for which a Path-BigBird model increased the micro *F*_1_ score by 1.44 points and the macro *F*_1_ score by 3.55 points. Overall, the results suggest that a Path-BigBird model with a vocabulary derived from well-curated and deidentified data is the best-performing model.

**CONCLUSION:**

The Path-BigBird pathology transformer model improves automated information extraction from pathology reports. Although Path-BigBird outperforms Clinical BigBird and HiSAN, these less computationally expensive models still have utility when resources are constrained.

## Teaser

Exploring advancements in cancer pathology research: Path-BigBird, a specialized transformer model, shows promise in extracting crucial tumor information from SEER cancer registry reports. Outperforming HiSAN and Clinical BigBird, it offers improved insights. A step toward enhanced pathology report analysis. #PathologyResearch #SEER #Transformers #LLM

## INTRODUCTION

In 2023 alone, an estimated 1.9 million new cancer cases will be diagnosed in the United States, and an estimated 609,000 cancer-related deaths will occur.^1^ To gain insights into cancer incidence and survival, the National Cancer Institute's (NCI's) SEER program collects information via 19 population-wide cancer registries as a primary source for unbiased population-level research. Pathology reports are the primary source of information used for phenotyping tumor cases. Traditionally, the cancer registrars manually review pathology reports to extract important phenotypic information from them.^2^ However, this manual review has contributed to a significant delay in NCI cancer incidence reporting.^3^ Recent advancements in deep learning (DL) and natural language processing (NLP) have made the near real time autoextraction of information from pathology reports an achievable goal.^4,5^ A Hierarchical Self-Attention Network (HiSAN) model is currently used in production across SEER registries to automatically extract information from approximately 25% of all records.^6^ Over the past 2 years, novel developments in the field have presented new opportunities for increasing the proportion of pathology reports that can be autocoded at high accuracy.

**CONTEXT**

**Key Objective**
To investigate the effectiveness of domain-specific transformer models for extracting information from cancer pathology reports, with a focus on tumor characteristics.
**Knowledge Generated**
A pathology transformer model called Path-BigBird was trained on 2.7 million pathology reports from SEER cancer registries. The research compares Path-BigBird with other methods, including Clinical BigBird and Hierarchical Self-Attention Network (HiSAN), across five information extraction tasks related to cancer pathology reports. The results demonstrate that Path-BigBird and Clinical BigBird outperform HiSAN in these tasks, with Path-BigBird excelling in the challenging tasks of subsite and histology extraction.
**Relevance *(J.L. Warner)***
Diagnostic pathology reports are crucial for accurate identification of disease (or lack thereof), yet due to the unstructured nature of these reports, they are not easily consumable by promising Clinical Decision Support systems as well as other research and registry reporting solutions. Additionally, such solutions could help many paper/fax-based referral pathology reports to be made easily available to the clinicians in a timely fashion.*
  *Relevance section written by *JCO CCI* Editor-in-Chief Jeremy L. Warner, MD, MS, FAMIA, FASCO.

The traditional classification model, which operates within a supervised DL framework, is characterized by a fixed architecture that accommodates only specific trained tasks. This rigid framework poses challenges for adapting the trained model to new prediction tasks. As a result, new models that use the same underlying data must be trained from scratch for each extraction task, thereby increasing the computational costs. Large-scale language models, which typically use the transformer model architecture, present a solution by extracting inherent patterns in text that can be used for additional supervised or unsupervised learning tasks beyond the initially trained task. Unsupervised learning with pretrained weights captures the underlying patterns in data but is naïve to an outcome. This method contrasts traditional DL supervised models, in which the weights are produced by using outcome-driven pattern recognition.^7,8^ The unsupervised nature of transformers has created an opportunity for general domain transformer models such as BERT^7^ and GPT,^9^ which are trained on general text corpora. The general domain transformers have created an accessible and accelerated framework for outcome-driven fine-tuning models for downstream tasks.

In recent years, the development of specialized biomedical and clinical transformers has expanded the applicability of transformer models to the health care domain. These models have been trained on various data sets, including PubMed abstracts, publicly available deidentified clinical notes from electronic health records (EHRs) such as MIMIC.^7,10-13^ By training on health care–specific data sets, clinical transformers can capture domain-specific patterns and terminology, thereby enabling them to perform tasks such as medical diagnosis, EHR analysis, clinical text classification, and entity recognition. Notably, the Clinical BigBird model has achieved state-of-the-art performance for longer sequences in a medical text by leveraging training with a sparse attention mechanism.^12^

In this study, we evaluate the effectiveness of transformer models for information extraction from pathology reports. Because a previous study^14^ found that a general domain transformer fine-tuned on pathology reports failed to outperform the benchmark HiSAN model, we revisit the question by developing a domain-specific transformer for pathology reports using the BigBird architecture. To delineate the effects of this domain-specific pathology transformer, Path-BigBird, we also assess the performance of Clinical BigBird, which was pretrained on a more general clinical notes corpus using the same transformer architecture. Accordingly, this study makes three contributions: (1) we introduce an approach for developing the pathology text–specific Path-BigBird model, (2) we test multiple versions of the Path-BigBird model against Clinical BigBird and the HiSAN baseline on a large pathology report data set, and (3) we identify cases in which Path-BigBird outperforms HiSAN and Clinical BigBird and evaluate the implications for future studies.

## METHODS

### Data Description

**FIG 1.**
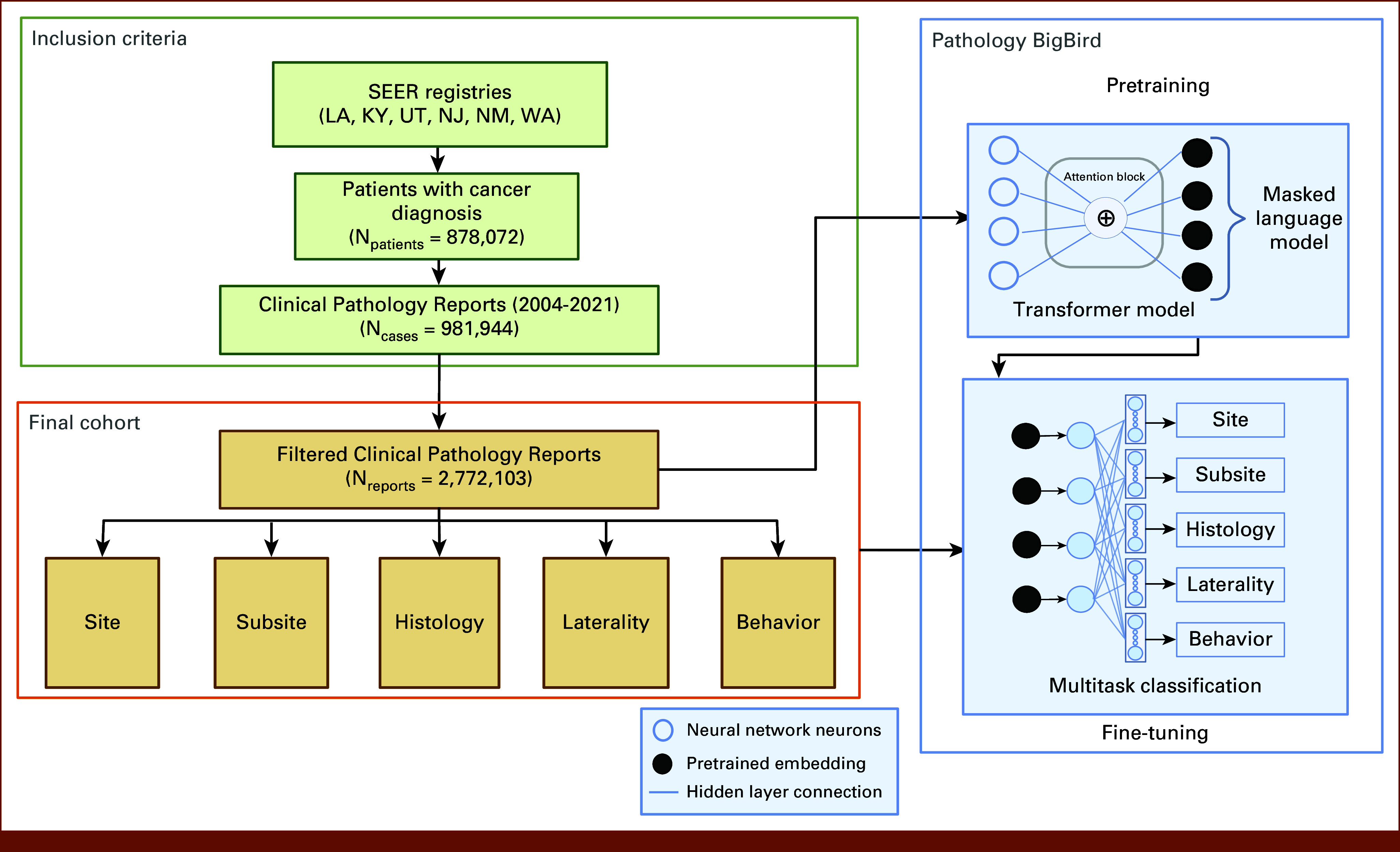
Flow diagram for Pathology BigBird (created with Bio-render). KY, Kentucky; LA, Louisiana; NJ, New Jersey; NM, New Mexico; UT, Utah; WA, Seattle.

Our data set is composed of electronic cancer pathology reports collected from six SEER Registries during a period from 2004 to 2021: Louisiana (LA), Kentucky (KY), Utah (UT), New Jersey (NJ), New Mexico (NM), and Seattle (WA). The combined data set has 2,772,103 pathology reports from 981,944 tumor cases and a total of 878,072 unique patients. Each registry collects cancer pathology reports from histology pathology laboratories, and each report describes the tumor and the date the report was generated. The cancer registrars abstract and code information from pathology reports with different disease classifications for oncology (ICD-O).^15^ These classifications are essential information and aid in cancer incidence tracking at the population level. We consider five classification tasks on the basis of annotated ICD-O classification: site, subsite, histology, laterality, and behavior (Fig 1). The site and subsite are based on 3-character and 5-character ICD-O-3 codes, respectively.^16^ Histology, laterality, and behavior tasks are the cancer characteristics.^17^ This research was approved by the DOE Institutional Review Board and determined to be exempt from informed consent: DOE000152.

We split the annotated pathology data set into three parts for model building: train, validation, and test. To reflect the real-world setting, the test data set was extracted by selecting the most recently diagnosed tumor cases per registry, which resulted the test data set to be 15% of the pathology data set for a total of 394,351 pathology reports from 169,663 tumor cases. The remaining records were randomly split into train (70%) and validation (15%). The distribution of records by training split across each cancer registry is shown in Table 1.

**TABLE 1.** Distribution of Reports, Cases, and Patients in the SEER Cancer Pathology Data Set by Training Split

Registry	Train		Validation		Test	
Seattle	629,284	32.12	134,551	34.12	111,749	26.69
New Jersey	369,441	18.86	78,874	20.00	74,742	17.85
Louisiana	363,345	18.55	77,620	19.68	76,305	18.22
Kentucky	361,981	18.48	76,920	19.51	80,588	19.25
Utah	140,025	7.15	30,298	7.68	28,435	6.79
New Mexico	94,935	4.85	20,478	5.19	22,532	5.38
Total pathology reports	1,959,011		418,739		394,351	
Total tumor cases	710,592		167,632		169,663	
Total patients	649,748		164,223		164,573	

### Outcome Labels: Five Tasks

The information extraction task consisted of the following categories: *site* with 70 classes, *subsite* with 324 classes, *behavior* with four classes, *histology* with 626 classes, and *laterality* with seven classes (Data Supplement, Table S1 shows number of classes across the training splits). Table 2 shows the top classes for each task and their prevalence across the training splits. The distribution of pathology reports across different classes highlights the imbalance of the data set. The information extraction labels were annotated by registrars for each tumor case. All pathology reports for the given tumor case are assigned the same labels. Data Supplement (Fig S1) shows the distribution of sequence length of pathology reports.

**TABLE 2.** Class-Wise Distribution of Reports for the Five Information Extraction Tasks Across Training Splits

Class Description	Train, % ↓		Validation, %		Test, %	
Task: Site (top 10 classes)						
C50: Breast	522,728	26.68	112,041	28.41	99,238	23.70
C34: Lung and bronchus	194,610	9.93	41,723	10.58	38,620	9.22
C44: Skin	145,628	7.43	30,884	7.83	33,569	8.02
C61: Prostate gland	144,102	7.36	30,733	7.79	34,529	8.25
C42: Blood, bone marrow, etc	136,112	6.95	28,322	7.18	26,814	6.40
C18: Large intestine, appendix	114,038	5.82	24,761	6.28	21,493	5.13
C77: Lymph nodes	76,923	3.93	16,047	4.07	13,942	3.33
C67: Urinary bladder	71,213	3.64	15,078	3.82	12,246	2.92
C54: Corpus uteri	68,604	3.50	15,000	3.80	15,742	3.76
C73: Thyroid gland	44,536	2.27	9,684	2.46	9,100	2.17
Task: Histology (top 10 classes)						
8140: Adenocarcinoma, NOS	401,016	20.47	85,872	21.78	92,590	22.11
8500: Duct carcinoma	357,780	18.26	77,172	19.57	81,454	19.45
8070: Squamous cell carcinoma, NOS	104,642	5.34	22,446	5.69	20,061	4.79
8720: Nevi and melanomas	71,606	3.66	15,179	3.85	15,724	3.76
8130: Papillary transitional cell carcinoma	53,876	2.75	11,333	2.87	9,142	2.18
8520: Lobular and other ductal carcinoma	49,235	2.51	10,376	2.63	10,079	2.41
8380: Endometrioid adenocarcinoma	48,090	2.45	10,695	2.71	11,387	2.72
8523: Lobular and other ductal carcinoma	37,059	1.89	8100	2.05	423	0.10
9680: ML, large B-cell, diffuse	36,699	1.87	7,544	1.91	7,074	1.69
8260: Papillary adenocarcinoma, NOS	29,823	1.52	6,490	1.65	6,652	1.59
Task: Subsite (top 10 classes)						
C504: Upper-outer quadrant of breast	168,297	8.59	35,998	9.13	33,260	7.94
C619: Prostate gland; prostate, NOS	144,102	7.36	30,733	7.79	34,529	8.25
C421: Bone marrow	133,485	6.81	27,706	7.03	26,514	6.33
C508: Overlapping lesion of breast	115,808	5.91	24,782	6.28	22,698	5.42
C341: Upper lobe, lung	99,007	5.05	21,057	5.34	19,467	4.65
C509: Breast, NOS	76,832	3.92	16,251	4.12	11,829	2.82
C541: Endometrium	65,673	3.35	14,363	3.64	15,502	3.70
C502: Upper-inner quadrant of breast	59,699	3.05	12,884	3.27	11,843	2.83
C343: Lower lobe, lung	52,371	2.67	11,450	2.90	10,980	2.62
C739: Thyroid gland	44,536	2.27	9,684	2.46	9,100	2.17
Task: Behavior						
3: Malignant, primary site (invasive)	1,758,656	89.77	354,615	89.92	375,512	89.68
2: Carcinoma in situ; noninvasive	185,374	9.46	36,660	9.30	39,978	9.55
0: Benign	12,207	0.62	2,553	0.65	2,688	0.64
1: Uncertain whether benign or malignant	2,774	0.14	523	0.13	563	0.13
Task: Laterality						
0: Not a paired site	957,571	48.88	203,856	51.69	196,932	47.03
1: Right: origin of primary	493,935	25.21	105,979	26.87	97,450	23.27
2: Left: origin of primary	471,125	24.05	100,992	25.61	92,652	22.13
9: Paired sites, but no information concerning laterality	16,951	0.87	3,561	0.90	3,308	0.79
4: Bilateral involvements at time of diagnosis	12,141	0.62	2,785	0.71	1,955	0.47
5: Paired sites: midline tumor (effective with January 1, 2010 dx)	5,945	0.30	1,297	0.33	1,860	0.44
3: Only one side involved, right or left origin unspecified	1,343	0.07	271	0.07	194	0.05

Abbreviations: ML, malignant lymphoma; NOS, not otherwise specified.

### Model Development and Evaluation

**FIG 2.**
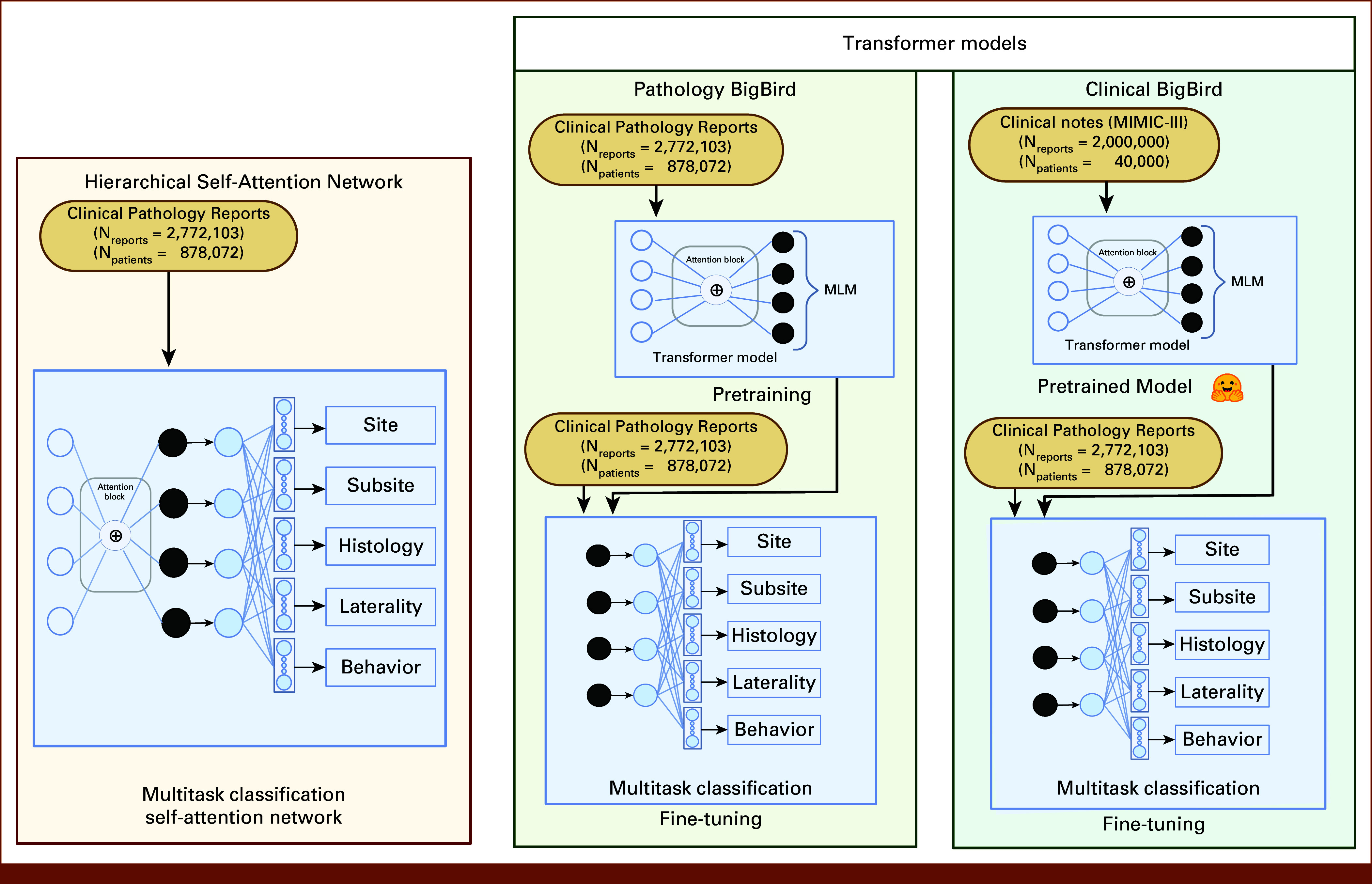
Comparison of models (created with Bio-render). MLM, masked language modeling.

In this study, we compare our novel pathology transformer (Path-BigBird) with two different model architectures: the current state-of-the-art classification model (HiSAN)^5^ and a clinical transformer (Clinical BigBird).^18^ As shown in Figure 2, the three model architectures have substantial differences. All three models use attention mechanisms.^8^ HiSAN is a multitask classification model, in which the feature extraction layer weights are trained based on the positive outcome on all five information extraction tasks.^5^ By contrast, the transformer models consist of a two-stage model building approach: an attention-based pretraining stage, followed by a fine-tuning stage with a classification setup. We compare the models in three categories: data tokenization, pretraining, and fine-tuning.

#### Data Tokenization

HiSAN and transformer models have different approaches to data tokenization. The HiSAN classification model uses a word2vec-based embedding, whereas the transformer model uses a subword tokenization approach. The word2vec approach is based on a fixed vocabulary, and this limits the ability to learn out-of-vocabulary words. Conversely, subword tokenization can handle out-of-vocabulary words by breaking them down into smaller units. We use two types of subword tokenizers for the transformer model: sentence piece (SP) tokenizer^19^ and word piece (WP) tokenizer.^20^

Clinical BigBird uses an SP tokenizer that has been pretrained on MIMIC-III EHRs with a vocabulary size of 50,358 (Fig 2). For Path-BigBird, we introduce three variations. First, Path-BigBird-WP uses a WP tokenizer trained on pathology reports with a vocabulary size of 32,000. Second, Path-BigBird-SP uses the same SP settings as Clinical BigBird, but the tokenizer is trained on pathology reports instead of MIMIC-III. Third, CV-Path-BigBird-SP uses the SP tokenizer from Clinical BigBird. The CV-Path-BigBird-SP model will help us understand how a vocabulary's source affects the model's performance.

#### Pretraining

One distinguishing factor of transformers from other traditional classification models is the pretraining stage, in which the learning of the model weights is not dependent on the clinical outcome. Typically, a classification model such as HiSAN does not have this pretraining stage. The transformer model's pretraining stage is based on an unsupervised task called masked language modeling.^7^ For pretraining, Path-BigBird uses the BigBird transformer model architecture on the basis of a sparse attention mechanism.^18^ In a standard dense attention mechanism, every token attends to every other token, and this can become computationally expensive for long sequences. Sparse attention reduces the number of tokens that each token attends to, thereby allowing for fast and efficient processing of long sequences and reducing the memory requirements without significantly compromising accuracy. Pretraining the pathology transformer models on SEER reports is intended to help capture and comprehend the unique language patterns within the pathology reports in the model (Fig 2).

Clinical BigBird is an off-the-shelf model pretrained on the MIMIC-III EHR data in a previous study.^12^ The comprehensive MIMIC-III data set comprises various clinical notes, including progress notes, radiology reports, and other relevant medical documents. By training on this diverse range of EHR notes, the clinical transformer learns the inherent patterns and structures within these types of medical records.

#### Fine-Tuning

Fine-tuning, or downstream training, involves taking a pretrained language model or a classification model and performing supervised learning on one or more classification tasks.^7^ The pretrained language model has already learned the patterns and relationships between words in a large corpus of text data, and fine-tuning the model on a specific task involves modifying the final layers of the model to predict the specific class labels for that task.

The classification model (HiSAN) and the fine-tuning task for the transformer models are designed as multitask classification models, specifically as five pathology information extraction tasks. Previous studies have demonstrated the advantages of multitask classification for pathology report classification.^5,21^

### Evaluation Metrics

To compare the different models, we use *F*_1_ micro scores and *F*_1_ macro scores as evaluation metrics on a holdout test data set. These metrics have been used in similar studies of pathology report classification.^5,22^ The *F*_1_ micro and *F*_1_ macro scores are calculated on all five tasks, which are being evaluated independently. The *F*_1_ micro measures the global performance of the model, and the *F*_1_ macro provides equal weight to each class and an overall evaluation of the model's performance across all classes. Confidence intervals were calculated using the normal approximation method,^23^ under the assumption that the accuracy of k models estimated with independent random draws from the test holdout data would produce accuracy scores that follow a normal distribution. We also measure the time taken to pretrain the models on the available high-performance computing resources, and this enables us to quantify the model's computational needs.

## RESULTS

Next, we present the performance results and a comparative analysis of HiSAN, Clinical BigBird, and Path-BigBird. Table 3 lists the micro and macro *F*_1_ scores, respectively, for HiSAN, Clinical BigBird, and Path-BigBird (see also Fig 2). The evaluation was conducted on the test split of the pathology data set, which consisted of 394,351 pathology reports (refer to Table 1). Notably, the HiSAN model was run to convergence, whereas the transformer fine-tuning tasks were run for a fixed number of epochs, which is potentially short of convergence.

**TABLE 3.** Macro and Micro Scores of Different Models With CI

Model Type	Model Name	Task	Micro With CI	Macro With CI
Classification	HiSAN	Site	92.82 (92.74 to 92.9)	69.81 (69.67 to 69.95)
Clinical transformer	Clinical BigBird		**93.26** (93.18 to 93.34)	**71.25** (71.11 to 71.39)
Pathology transformer	Path-BigBird-SP		92.69 (92.61 to 92.77)	69.34 (69.20 to 69.48)
	Path-BigBird-WP		93.01 (92.93 to 93.09)	70.72 (70.58 to 70.86)
	CV-Path-BigBird-SP		92.87 (92.79 to 92.95)	70.4 (70.26 to 70.54)
Classification	HiSAN	Subsite	70.71 (70.57,70.85)	34.06 (33.91 to 34.21)
Clinical transformer	Clinical BigBird		72.45 (72.31 to 72.59)	34.22 (34.07 to 34.37)
Pathology transformer	Path-BigBird-SP		71.46 (71.32 to 71.6)	34.21 (34.06 to 34.36)
	Path-BigBird-WP		**72.53** (72.39 to 72.67)	35.13 (34.98 to 35.28)
	CV-Path-BigBird-SP		72.34 (72.2 to 72.48)	**35.76** (35.61 to 35.91)
Classification	HiSAN	Laterality	92.09 (92.01 to 92.17)	53.83 (53.67 to 53.99)
Clinical transformer	Clinical BigBird		**92.78** (92.7 to 92.86)	**56.66** (56.51 to 56.81)
Pathology transformer	Path-BigBird-SP		92.25 (92.17 to 92.33)	54.92 (54.76 to 55.08)
	Path-BigBird-WP		92.61 (92.53 to 92.69)	56.03 (55.88 to 56.18)
	CV-Path-BigBird-SP		92.56 (92.48 to 92.64)	56.51 (56.36 to 56.66)
Classification	HiSAN	Histology	79.25 (79.12 to 79.38)	33.22 (33.07 to 33.37)
Clinical transformer	Clinical BigBird		77.46 (77.33 to 77.59)	33.49 (33.34 to 33.64)
Pathology transformer	Path-BigBird-SP		79.36 (79.23 to 79.49)	32.07 (31.92 to 32.22)
	Path-BigBird-WP		80.66 (80.54 to 80.78)	34.42 (34.27 to 34.57)
	CV-Path-BigBird-SP		**80.69** (80.57 to 80.81)	**37.04** (36.89 to 37.19)
Classification	HiSAN	Behavior	97.57 (97.52 to 97.62)	88.92 (88.82 to 89.02)
Clinical transformer	Clinical BigBird		97.85 (97.8 to 97.89)	91.05 (90.96 to 91.14)
Pathology transformer	Path-BigBird-SP		97.69 (97.64 to 97.74)	89.12 (89.02 to 89.22)
	Path-BigBird-WP		**97.92** (97.88 to 97.96)	90.64 (90.55 to 90.73)
	CV-Path-BigBird-SP		97.89 (97.85 to 97.93)	**91.06** (90.97 to 91.15)

Abbreviation: HiSAN, Hierarchical Self-Attention Network. Bold values indicate the best performing model scores for each task.

When comparing the transformer models with the HiSAN models, the transformer models outperform HiSAN in both micro and macro *F*_1_ scores across all tasks. The best-performing model is listed in bold font within the tables. For all five tasks, the best-performing model is a transformer. Notably, when comparing the clinical transformer with the pathology transformers, Clinical BigBird has better micro *F*_1_ scores for the *site* task (with 93.26%) and the *laterality* task (with 92.78%). By contrast, the pathology transformer models excel in the *subsite* (with 72.53%), *histology* (with 80.69%), and *behavior* (with 97.92%) tasks. The superior performance of the pathology transformer models in the *subsite* and *histology* categories is not surprising because these two tasks involve the most classes and are, therefore, more challenging. Because the pathology transformer models were pretrained and fine-tuned on pathology reports, they can better capture subtle language patterns to differentiate between the various classes.

When comparing the models' macro *F*_1_ scores, the CV-Path-BigBird-SP model and Clinical BigBird are the best-performing models. The CV-Path-BigBird-SP achieves the highest *F*_1_ macro score on the most difficult tasks: *subsite* with 35.76% and *histology* with 37.04%. The macro *F*_1_ performance on *histology* had the highest jump of 3%-4% compared to the other models. The results indicate that vocabulary from deidentified EHRs benefits pathology reports. EHR-based vocabulary is more compact but diverse during pretraining, which leads to better generalization of the model and improved performance on pathology data extraction tasks (Data Supplement, Table S2 displays micro and macro *F*_*1*_ scores for all tasks across different sequence length groups, providing insight into generalization across varying sequence lengths).

Table 4 shows the class-wise accuracy of CV-Path-BigBird-SP for the different tasks, highlighting accuracy disparity among classes, especially for the difficult tasks: subsite and histology. In reference to subsite and histology, it has come to our attention that the least prevalent categories exhibited an *F*_1_ score of zero for 12% and 36% of the subsite and histology classes, respectively. This finding is a contributing factor to the previously discussed low macro *F*_1_ score. We observe a general decrease in *F*_1_ score as prevalence decreases for site, laterality, and behavior tasks (detailed class-wise accuracy is reported in the Data Supplement, Tables S3-S7).

**TABLE 4.** Class Wise Accuracy Across Tasks on Test Split

Site			Subsite			Histology		
Label	*F*_1_ Score	Reports↓	Label	*F*_1_ Score	Reports↓	Label	*F*_1_ Score	Reports↓
C50	99.33	99,267	C619	98.96	34,539	8140	90.34	92,608
C34	94.98	38,626	C504	67.23	33,271	8500	92.14	81,483
C61	99.05	34,539	C421	93.89	26,516	8070	82.22	20,066
C44	98.30	33,573	C508	56.58	22,706	8720	81.32	15,727
C42	94.42	26,816	C341	76.07	19,469	8380	90.38	11,388
C18	93.01	21,496	C541	95.26	15,504	8520	85.66	10,080
C54	95.39	15,744	C502	63.22	11,850	8130	86.76	9,142
C77	81.92	13,945	C509	33.99	11,829	8743	75.30	7,434
C67	94.98	12,248	C343	69.38	10,983	9680	81.53	7,076
C73	98.46	9,102	C445	93.16	10,099	8260	85.67	6,652
Least prevalent site classes			39 of least prevalent subsite classes with zero to six reports had zero *F*_1_ score			227 of least prevalent histology label with zero to six reports had zero *F*_1_ score		
C08	53.67	199						
C13	40.13	167						
C12	60.77	160						
C37	67.09	154						
C63	73.54	127						
C47	37.50	108						
C14	18.44	90						
C33	43.48	33						
C58	68.75	19						

Laterality			Behavior		
Label	*F*_1_ score	Reports↓	Label	*F*_1_ score	Reports↓
0	96.02	196,973	3	98.84	354,690
1	90.48	97,475	2	89.18	36,673
2	90.85	92,672	0	89.18	2,554
9	14.16	3,308	1	80.74	523
4	36.84	1,956			
5	67.18	1,862			
3	00.00	194			

A model's building time is an essential metric because transformers are compute-intensive models owing to their complex architecture and the many parameters in the model. Path-BigBird is the most computationally expensive model, whereas Clinical BigBird uses off-the-shelf embeddings, and the HiSAN does not have a pretraining stage. For fine-tuning, Path-BigBird and Clinical BigBird follow identical processes, resulting in similar time requirements (please refer to the Data Supplement, Table S8, which lists the model training times). However, it is worth noting that the fine-tuning process for the transformer models takes longer because of the need to update the weights of both the pretraining and fine-tuning stages. Despite the computational demands, these findings highlight the importance of transformers for capturing complex patterns and relationships, thereby facilitating enhanced performance in pathology information extraction tasks.

## DISCUSSION

In this study, we describe the effectiveness of using large language models (LLMs) in the clinical pathology domain. The study and development of domain-specific transformers has become increasingly important with the widespread availability and ease of use of pretrained general domain language models. These general domain transformers, although powerful in various language tasks, often lack the specialized medical knowledge required for accurate decision making and meaningful insights in clinical data.^24,25^ We show that transformer models perform better than our previously published HiSAN model on the five pathology information extraction tasks.^5^

Our results suggest that customizing transformer models for specific clinical domains (eg, oncology) is important. Generic off-the-shelf models are generally trained using general clinical data and may fail to capture the nuances and intricacies unique to oncology. Moreover, although using general clinical transformers to extract information from pathology reports may be an effective strategy in a computationally constrained environment, limitations still exist in their ability to capture the subtle language nuances specific to pathology. These subtle nuances play a crucial role in distinguishing classes in more challenging tasks, such as *histology* and *subsite*. This highlights the need for domain-specific models to achieve the accuracy required for deployment in health care applications. Thus, although general or clinical off-the-shelf models can be useful, the development of specialized models increases accuracy in health care contexts.

Notably, our study also revealed the importance of the model's tokenizer and vocabulary to the pathology report classification. First, we found that the improved performance of the transformer models can be partially attributed to using subword tokenizers because they can handle out-of-vocabulary words by breaking them down into smaller units. Second, we found that CV-Path-BigBird-SP has superior performance because it uses a cleaner, deidentified vocabulary compared with other pathology transformers. This observation highlights the significant impact of vocabulary definition on the performance of language models. Surprisingly, this EHR-derived vocabulary, although created from a clinical context rather than specifically tailored for pathology, yielded better results (in terms of macro *F*_1_ score) than the pathology-specific vocabulary. This raises an important question about the reliance on LLMs to comprehend and process data. By ensuring standardized and privacy-preserving data, we can enhance model performance. This highlights the trade-off between domain specificity and data cleanliness when training language models for sensitive clinical tasks. The success of CV-Path-BigBird underscores the value of thorough data preparation for accurate outcomes in clinical NLP. It also encourages further exploration of data cleaning and deidentification techniques to facilitate data privacy and improved performance in health care applications.

Recent advances in LLMs have piqued the interests of researchers and clinicians in the potential benefits of artificial intelligence (AI) for health care. Our previous projects have shown that AI can be used to improve the speed and accuracy of disease reporting at the national scale.^26^ With this project, we show that the recent advances in LLMs can be adapted to improve the performance of current AI tools. We have demonstrated the power of using a population-wide repository of pathology reports to train a domain-specific transformer. In doing so, we have identified potential opportunities for improving and expanding this research. First, future studies should quantify the data and algorithmic biases that may unequally affect marginalized populations. Although our data are broadly representative of the US population,^27,28^ the level of detail and semantics may differ by race, socioeconomic status, and so on. Second, future studies should explore more advanced ways to quantify the uncertainty in each classification.^29^ The work presented here is a glimpse of the upcoming AI-powered paradigm shift in cancer care. Although we must be cautious, these tools hold the potential to significantly improve patient care.

Transformer models have emerged as powerful tools for information extraction tasks in cancer pathology reports. These models, including clinical transformers, can capture complex patterns and correlations between words in clinical text. The availability of pretrained language models has facilitated the development of domain-specific clinical downstream tasks. However, when pretraining, we must consider the domain gap between generic text and domain-specific text to ensure reliable performance. Using domain-specific text can contribute to the accurate extraction of relevant information from cancer pathology reports, thereby facilitating improved clinical decision making and patient care. For future work, we plan to further test the model's generalizability and reusability by extending the tasks to other clinically useful tasks, such as biomarker extraction and identification of malignant and metastatic disease.
